# Supplementary material for: Macroscale cortical organization and a default-like apex transmodal network in the marmoset monkey
Source: Nat Commun. 2019 Apr 29;10:1976. doi: 10.1038/s41467-019-09812-8 (PMC6488644; doi:10.1038/s41467-019-09812-8)
Supplement: Supplementary file 1 — Supplementary Information [file 41467_2019_9812_MOESM1_ESM.pdf]

Macroscale Cortical Organization and a Default-Like Apex Transmodal Network in the Marmoset Monkey

Buckner et al.

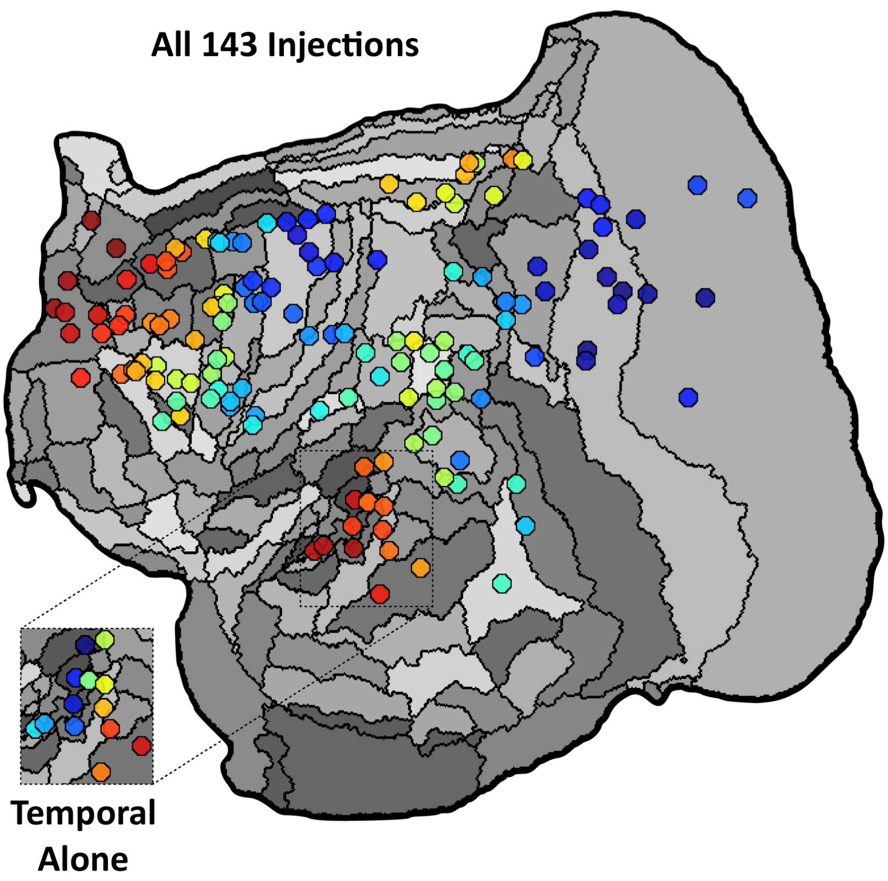

Supplementary Figure 1. Transmodal Association Cortex Is Robustly Distinguished From Sensory Hierarchies When Analyzed by Unbiased Factor Analysis. Displayed are all 143 injection locations from the Marmoset Brain Architecture Project archive as of August, 2018 color coded based on their association with the major factor that distinguishes projection patterns in the marmoset cortex. The main figure presents the rank order of all injections, derived from diffusion map embedding of a similarity matrix calculated across flatmap connectivity patterns. While the first factor differentiated visual from somatosensory/motor areas, here we show the second factor corresponding with the spectrum from primary to apex areas. Injections displayed with red-yellow colors show the strongest weighting, while blue colors the opposite. The inset image presents a secondary analysis restricted to temporal lobe injections, in which the first factor demonstrated the gradient between auditory regions and areas TPO, PGa/IPa, and TE3. Code is available at https://github.com/margulies/marmoset.
